# *O*-acetylation controls the glycosylation of bacterial serine-rich repeat glycoproteins

**DOI:** 10.1074/jbc.RA120.016116

**Published:** 2021-01-09

**Authors:** Ravin Seepersaud, Alexander C. Anderson, Barbara A. Bensing, Biswa P. Choudhury, Anthony J. Clarke, Paul M. Sullam

**Affiliations:** 1 Department of Medicine, Division of Infectious Diseases, San Francisco Veteran Affairs Medical Center, and the Department of Medicine, University of California, San Francisco, California, USA; 2 Department of Molecular and Cellular Biology, University of Guelph, Guelph, Ontario, Canada; 3 GlycoAnalytics Core, University of California, San Diego, San Diego, California, USA; 4 Department of Chemistry and Biochemistry, Wilfrid Laurier University, Waterloo, Ontario, Canada

**Keywords:** accessory, glycoprotein:glucosylation, *O*-acetylation, *Streptococcus gordonii*, aSec, accessory Sec, Asp2, aSec protein 2, BR, binding region, DPBS, Dulbecco's PBS, GspB, gordonii surface protein, GtfAB, glycosyltransferase AB complex, Gtfs, glycosyltransferases, PMAA, partially methylated alditol acetate, SRR, serine-rich repeat

## Abstract

The serine-rich repeat (SRR) glycoproteins of gram-positive bacteria are a family of adhesins that bind to a wide range of host ligands, and expression of SRR glycoproteins is linked with enhanced bacterial virulence. The biogenesis of these surface glycoproteins involves their intracellular glycosylation and export *via* the accessory Sec system. Although all accessory Sec components are required for SRR glycoprotein export, Asp2 of *Streptococcus gordonii* also functions as an *O*-acetyltransferase that modifies GlcNAc residues on the SRR adhesin gordonii surface protein B (GspB). Because these GlcNAc residues can also be modified by the glycosyltransferases Nss and Gly, it has been unclear whether the post-translational modification of GspB is coordinated. We now report that acetylation modulates the glycosylation of exported GspB. Loss of *O*-acetylation due to *aps2* mutagenesis led to the export of GspB glycoforms with increased glucosylation of the GlcNAc moieties. Linkage analysis of the GspB glycan revealed that both *O*-acetylation and glucosylation occurred at the same C6 position on GlcNAc residues and that *O*-acetylation prevented Glc deposition. Whereas streptococci expressing nonacetylated GspB with increased glucosylation were significantly reduced in their ability to bind human platelets *in vitro*, deletion of the glycosyltransferases *nss* and *gly* in the *asp2* mutant restored platelet binding to WT levels. These findings demonstrate that GlcNAc *O*-acetylation controls GspB glycosylation, such that binding *via* this adhesin is optimized. Moreover, because *O*-acetylation has comparable effects on the glycosylation of other SRR adhesins, acetylation may represent a conserved regulatory mechanism for the post-translational modification of the SRR glycoprotein family.

The serine-rich repeat (SRR) glycoproteins are a family of adhesins found on the surface of many gram-positive bacteria. Originally identified in oral streptococci, they have subsequently been found in several other genera and numerous species. Their expression has been directly correlated with enhanced bacterial virulence in a number of disease settings, such as endocarditis, pneumonia, and meningitis (1, 2, 3, 4, 5). More recently, the SRR glycoproteins have been found in nonpathogenic commensal bacteria, promoting bacterial adherence to epithelial surfaces of the vaginal mucosa, gastrointestinal tract, lung epithelia, and oral surfaces, as well as mediating biofilm formation (6, 7). These findings underscore the importance of the SRR glycoproteins as bacterial-host attachment factors.

The domain organization of all the SRR glycoproteins follows a conserved architecture (Fig. 1*A*). The N-terminus contains an extended signal peptide that is necessary for preprotein targeting and export *via* the accessory Sec (aSec) system (8). This region is followed by two SRR domains (SRR1 and SRR2) that are extensively glycosylated and that flank a ligand binding region (BR). The structure of the BR domain can significantly vary between species, resulting in binding to a variety of host targets exposed on mucosal and epithelial surfaces, including sialoglycans, fibrinogen, and keratin-10 (9, 10, 11, 12, 13, 14). A LPxTG sequence at the C-terminus covalently attaches the SRR glycoprotein to the cell wall (15).

**Figure 1:**
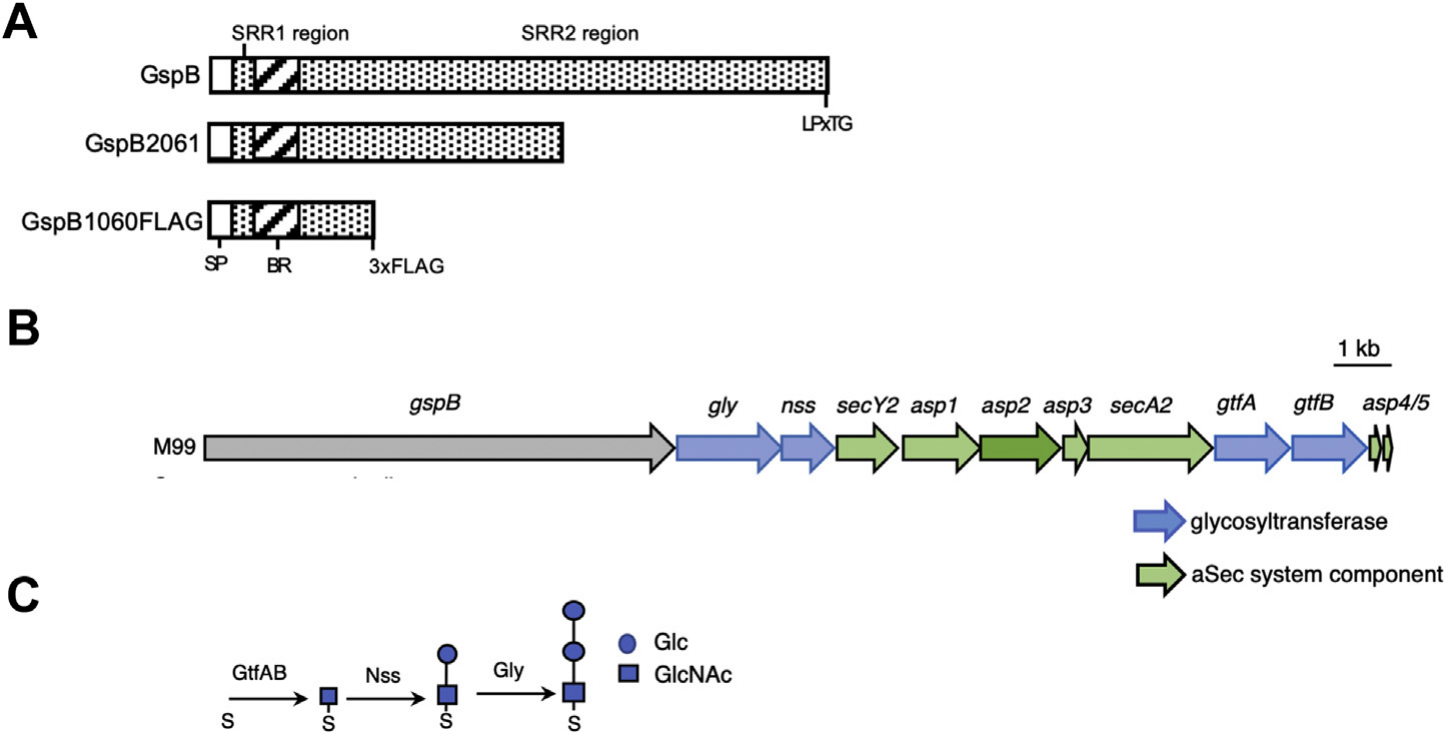
**Gordonii surface protein (GspB) and accessory Sec operon.***A*, the schematic of WT GspB and the recombinant constructs, GspB2061 and GspB1060FLAG. *B*, the *gspB-aSec* operon. Components of the accessory aSec (aSec) system are shown in *green*, and glycosyltransferases are in *blue*. *C*, sequential steps in the modification of GspB by GtfAB, Nss, and Gly. GspB glycan structures were determined previously by MALDI-TOF mass spectrometry analysis (27). SP is the signal peptide; SRR1 and SRR2 are the serine-rich repeat regions that undergo post-translational modification; Asp, accessory Sec protein; BR, ligand-binding region; GtfAB, glycosyltransferase complex; LPxTG, cell wall anchoring domain.

The SRR adhesins are glycosylated by a number of glycosyltransferases (Gtfs) encoded within the *gspB-aSec* operon (Fig. 1*B*). A two-protein glycosyltransferase complex (GtfAB) *O*-glycosylates the SRR protein by transferring GlcNAc to serine and threonine residues within the SRR domains (16, 17), (Fig. 1*C*). The remaining Gtfs further extend the glycan by adding other sugar moieties to the GlcNAc core (18, 19, 20, 21). The number and types of these additional Gtfs vary considerably between species, as do the resulting glycan structures (6, 20, 22).

The SRR glycoprotein gordonii surface protein (GspB) of *Streptococcus gordonii* is among the most extensively studied SRR glycoproteins. GspB is a Siglec-like adhesin that binds human platelets *in vitro* through its interaction with sialyl T-antigen moieties on the platelet GPIbα receptor (23, 24). This interaction is thought to contribute to the pathogenesis of infective endocarditis, where the expression of GspB has been associated with increased virulence in animal models of this disease (1). Biochemical and genetic studies of GspB biogenesis have identified two additional Gtfs (Nss and Gly) that further modify the GlcNAc core of GspB (17, 25) (Fig. 1*C*). Nss first transfers Glc to GlcNAc, allowing Gly to transfer a second Glc residue, thereby generating an *O*-linked trisaccharide (Fig. 1*C*).

Glycosylation is in part required for GspB stability, in that nonglycosylated GspB variants (produced by Δ*gtfA* or Δ*gtfB* strains) are rapidly degraded *in vivo* as compared with glycosylated forms (26). In addition, deletion of the secondary *gtf* genes can result in the export of stable but altered SRR glycoforms with impaired bacterial binding to their host substrates. Deletion of *nss* or *gly* in *S. gordonii* M99 results in WT levels of GspB surface expression but is associated with slightly reduced bacterial binding to human platelets (25, 27). Comparable results have been seen in *Streptococcus agalactiae*, where deletion of six Gtfs (*gtfC-gtfH*) that modify the adhesin Srr1 significantly reduced bacterial binding to human lung and intestinal epithelial cells (4). Similarly, individual deletion of the *gtfs* (*gtf3*-*gly*) that modify Fap1 of *Streptococcus parasanguinis* led to the formation of biofilms with altered biomass (21). Thus, maintaining a specific SRR glycoform appears to be essential for optimal SRR glycoprotein function.

In addition to glycosylation, we recently found that GlcNAc residues on GspB can undergo *O*-acetylation (27). This modification is carried out by the aSec protein 2 (Asp2), a member of the aSec system, a specialized transporter that mediates the export of SRR adhesins (8, 28). Acetylation of the GspB glycan was shown to be coupled to aSec transport, such that rerouting the substrate to the general Sec system prevented GlcNAc modification (27). Thus, the post-translational modification and export of SRR adhesins appear to be coordinated steps in the biogenesis of these glycoproteins, such that the adhesin is modified to maximize its binding properties.

Asp2 homologues are found in all aSec systems, and *O*-acetylated GlcNAc residues have been described on the SRRs of *S. agalactiae* and *Streptococcus salivarius* (29, 30). Analysis of glycopeptide fragments derived from exported GspB and Srr1 showed that *O*-acetylation and glucosylation of the GlcNAc core only occurred on a fraction of GlcNAc residues throughout the SRR domains (17, 27, 29). The cause of this glycosylation heterogeneity has been unclear. Of note, however, *S. gordonii* expressing an Asp2 mutant lacking acetyltransferase activity exported a GspB glycoform of increased mass, as compared with the WT glycoprotein, suggesting a significant change in its glycan composition (31). Moreover, isogenic variants of *S. gordonii* expressing this altered GspB glycoform showed reduced binding to human platelets (27). However, the precise changes in SRR glycan composition of this glycoform were unknown. We now show that a loss of GlcNAc *O*-acetylation leads to increased glycosylation of GspB by Nss and Gly and that this excessive addition of glycan on the SRR regions impairs GspB-mediated binding to platelets. The finding that GlcNAc *O*-acetylation modulates GspB glycosylation indicates that *O*-acetylation is a novel control mechanism that regulates both the extent of glycosylation and the binding properties of the SRR adhesins.

## Results

### GlcNAc *O*-acetylation limits glucose deposition by Nss and Gly on exported GspB

Asp2 acetylates GlcNAc moieties on GspB, and the loss of *O*-acetylation leads to the export of altered GspB glycoforms of increased mass, as compared with the WT (27, 31). This change in mass is less evident when *nss* and *gly* are deleted, suggesting that *O*-acetylation may interfere with Nss and Gly-mediated glucosylation of GspB. To test this possibility, we evaluated the changes in glycosylation of two truncated variants of GspB (GspB1060FLAG and GspB2061) containing 417 aa and 1428 aa of the SRR2 domain, respectively (Fig. 1*A*). Each protein was expressed in isogenic variants of *S. gordonii* M99 with different Gtf and *O*-acetylation backgrounds (Table 1).

**Table 1:** Bacterial strains and plasmids

Strain or plasmid	Genotype or description	Reference
*S. gordonii*		
M99	Endocarditis causing parental strain	(51)
PS1225	M99 expressing gspB736FLAG	(26)
PS3539	PS1225 *asp2*^S362A^	(27)
PS921	M99 expressing gspB1060FLAG	(26)
PS3874	PS921 *Δgly;* Spec^R^	This study
PS3318	PS921 *Δgly-nss*; Spec^R^	(27)
PS3541	PS921 asp2^S362A^	(27)
PS3875	PS3541 *Δgly* asp2^S362A^; Spec^R^	This study
PS3542	PS3541 *Δgly-nss* asp2^S362A^; Spec^R^	(27)
PS1064	PS921 *ΔgtfA;* Spec^R^	(27)
PS497	M99 *gspB* ::pB2061Hi s6; Cm^R^	(52)
PS612	PS497 *Δgly;* Spec^R^	This study
PS3876	PS497 *Δgly-nss*; Spec^R^	This study
PS3877	PS497 *asp2*^*S362A*^	This study
PS3878	PS497 *Δgly* asp2^S362A^	This study
PS3879	PS497 *Δgly-nss* asp2^S362A^	This study
PS666	M99 *ΔgtfA;* Spec^R^	(25)
PS3319	M99 *Δgly-nss;* Spec^R^	(27)
PS3536	M99 asp2^S362A^	(27)
PS3880	M99 *Δgly-nss* asp2^S362A^; Spec^R^	This study
*S. aureus*		
PS767	ISP479C *sraP::ermB*	(48)
PS1280	ISP479C asp2::spec	(48)
PS3855	PS1280 (pKS80); Erm^R^	This study
PS3856	PS1280 (pKS _H6sa_Asp2); Erm^R^	This study
PS3857	PS1280 (pKS _H6sa_Asp2^S368A^); Erm^R^	This study
*S. agalactiae*		
A909	Serotype Ia clinical isolate	(43)
PS3204	*A909Dsrr1*	(10)
PS3860	*A909Dasp2*	This study
PS3862	PS3860 (pDE _H6_gbsAsp2)	This study
PS3833	PS3860 (pDE _H6_gbsAsp2^S346A^)	This study
*S. mitis*		
SF100	endocarditis causing parental strain	(53)
PS3397	SF100 expressing SRRFLAG	This study
PS3870	*PS3397Dasp2*	This study
PS3871	*PS3397Dasp2* (pDE _H6_smAsp2)	This study
PS3872	*PS3397Dasp2* (pDE _H6_smAsp2^S362A^)	This study
*E. coli*		
TOP10	Host cell for cloning	
BL21	Host cell for protein expression	
Plasmids		
pET28Nss _H6_	*E. coli* expression vector encoding Nssh6; Kan^R^	This study
pC326	*E. coli* vector with MCS::cat cassette; Cm^R^	This study
pCasp2-KO[A909]	Group B streptococcus A909 *asp2 KO vector*; Cm^R^	This study
pS326	*E. coli* vector with MCS::spec cassette; Spec^R^	This study
pSasp2-KO[SF100]	*S. mitis* SF100 *asp2* KO vector; Spec^R^	This study
pKS80	*S. aureus* expression vector; Erm^R^	(47)
pKS_H6sa_Asp2	*S. aureus* expression vector encoding _H6sa_Asp2; Erm^R^	This study
pKS_H6sa_Asp2^S368A^	*S. aureus* expression vector encoding _H6sa_Asp2^S368A^; Erm^R^	This study
pDE123	Streptococcal expression vector; Erm^R^	(44)
pDE_H6_gbsAsp2	Streptococcal expression vector encoding _H6_gbsAsp2; Erm^R^	This study
pDE_H6_gbsAsp2^S346A^	Streptococcal expression vector encoding _H6_gbsAsp2^S346A^; Erm^R^	This study
pDE_H6_smAsp2	Streptococcal expression vector encoding _H6_smAsp2; Erm^R^	This study
pDE_H6_smAsp2^S362A^	Streptococcal expression vector encoding _H6_smAsp2^S362A^; Erm^R^	This study

Cm^R^, chloramphenicol resistant; Erm^R^, erythromycin resistant; Spec^R^, spectinomycin resistant; Kan^R^, kanamycin resistant.

**Figure 2:**
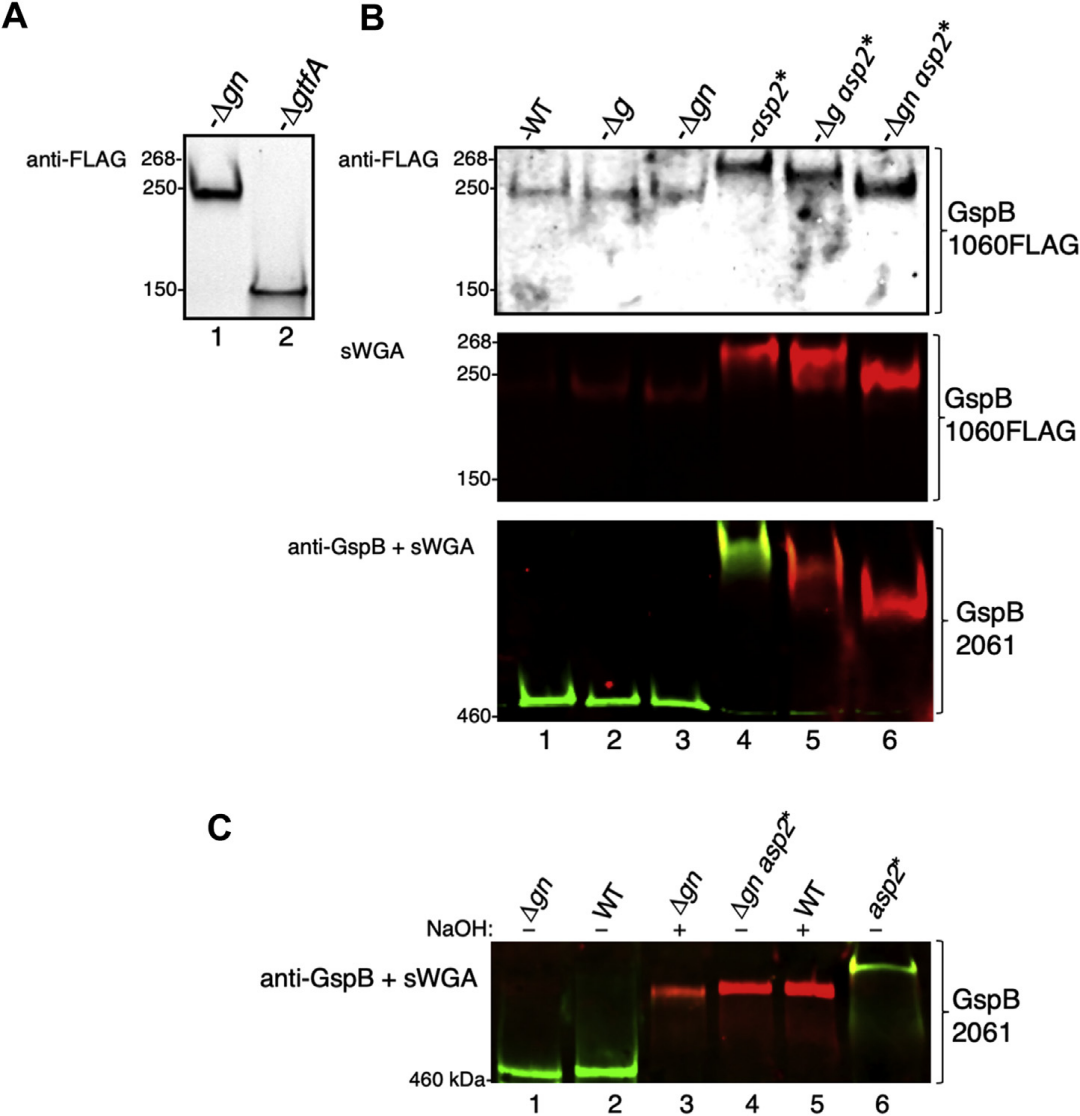
**Loss of GlcNAc *O*-acetylation leads to an increase in glucosylation of exported GspB by Nss and Gly.***A* and *B*, western blot analysis of GspB1060FLAG and GspB2061 exported by isogenic variants of M99 containing a deletion in *gtfA* (Δ*gtfA*), *gly* (Δ*g*), *gly* and *nss* (Δ*gn*), and/or an *asp2*^S362A^ mutation (*asp2*∗). Samples of culture media were separated by SDS-PAGE and probed with anti-FLAG antibodies and biotinylated sWGA, to detect levels of GspB and core GlcNAc, respectively. Exported GspB2061 was simultaneously probed with biotinylated sWGA (700-nm channel red) and anti-GspB polyclonal antisera raised against the WT GspB glycan (800-nm channel-green in overlay). *C*, saponification of GspB glycoforms. Exported GspB2061 glycoforms were treated with 100-mM NaOH at 37 °C for 1 h, to remove O-acetyl moieties. Changes to the GspB glycan were assessed by GspB2061 binding to both sWGA (700-nm channel *red*) and anti-GspB (800-nm channel *green* in overlay) glycan probes. GspB, gordonii surface protein.

We first assessed the individual contribution of each Gtf to the composition of the glycan on exported GspB. Deletion of *gtfA* resulted in a large reduction in the mass of GspB1060FLAG (Fig. 2*A*, lane 1 *versus* 2), demonstrating that GlcNAc is a major component of the GspB glycan. In contrast, the deletion of *gl*y (Δ*g*) or *gly* and *nss* (Δ*gn*) produced only minor changes in the electrophoretic mobilities of GspB1060FLAG and GspB2061, as compared with the WT glycoforms (Fig. 2*B*, lane 1 *versus* lanes 2 and 3), indicating a low abundance of Glc in the WT glycan.

To examine the effect of *O*-acetylation upon GspB glycan composition, we introduced an *asp2*^S362A^ mutation into the genome of M99, producing a catalytic site mutation that abolishes GlcNAc *O*-acetyltransferases activity (27). This mutation was associated with an increase in apparent mass for both GspB1060FLAG and GspB2061 as compared with the WT strain (Fig. 2*B*, lane 1 *versus* lane 4). The increase in mass was most evident with the longer GspB2061 variant, likely reflecting the larger number of serine residues available for modification. In addition, binding of sWGA to both GspB variants was increased, consistent with our previous observation that this lectin has higher affinity for GlcNAc, as compared with *O*-acetylated GlcNAc (Fig. 2*B*, lane 1 *versus* lane 4) (27). Deletion of *gly*, or *gly* and *nss*, in an *aps2*^S362A^ mutant resulted in a progressive reduction in the apparent mass of GspB (Fig. 2*B*, lane 4 *versus* lanes 5 and 6), further indicating that the increased mass of GspB associated with the loss of GlcNAc *O*-acetylation is at least in part due to increased Glc deposition by Gly and Nss on the GspB glycan.

### *O*-acetylation alters the electrophoretic mobility of GspB

GspB2061 modified with *O*-acetylated GlcNAc (exported by M99Δ*gn*) migrated with an apparent mass that was less than that of GspB2061 modified by nonacetylated GlcNAc (exported by M99Δ*gn asp2*^S362A^) (Fig. 2*B*, lane 3 *versus* lane 6). Because an acetyl group will add 43 Da to the mass of GlcNAc, it was unclear why acetylation would lead to an apparent decrease in mass. To address this issue, we exposed different glycoforms of GspB2061 to mild-base ester hydrolysis (saponification), which removes *O*-acetyl groups (27). Saponification of WT GspB2061 increased the apparent mass of the glycoprotein to that of nonacetylated GspB2061 (exported from M99Δ*gn asp2*^S362A^) (Fig. 2*C*, lanes 2 *versus* lane 5). Saponification of GspB2061 modified with GlcNAc alone produced a similar increase in mass (Fig. 2*C*, lane 3). These findings confirm that *O*-acetylation of GspB reduces its apparent mass on SDS-PAGE, which could be due to changes in hydrophobicity or viscosity (32). Importantly, we also found that saponified WT GspB2061 migrated at a lower mass than nonacetylated GspB2061 (exported by M99 *asp2*^S362A^) (Fig. 2*C*, lane 5 *versus* lane 6), suggesting that the increased mass of the latter is also due to increased glucose deposition by Nss and Gly.

### *O*-acetyl moieties and glucose residues modify core GlcNAc at the same position

**Figure 3:**
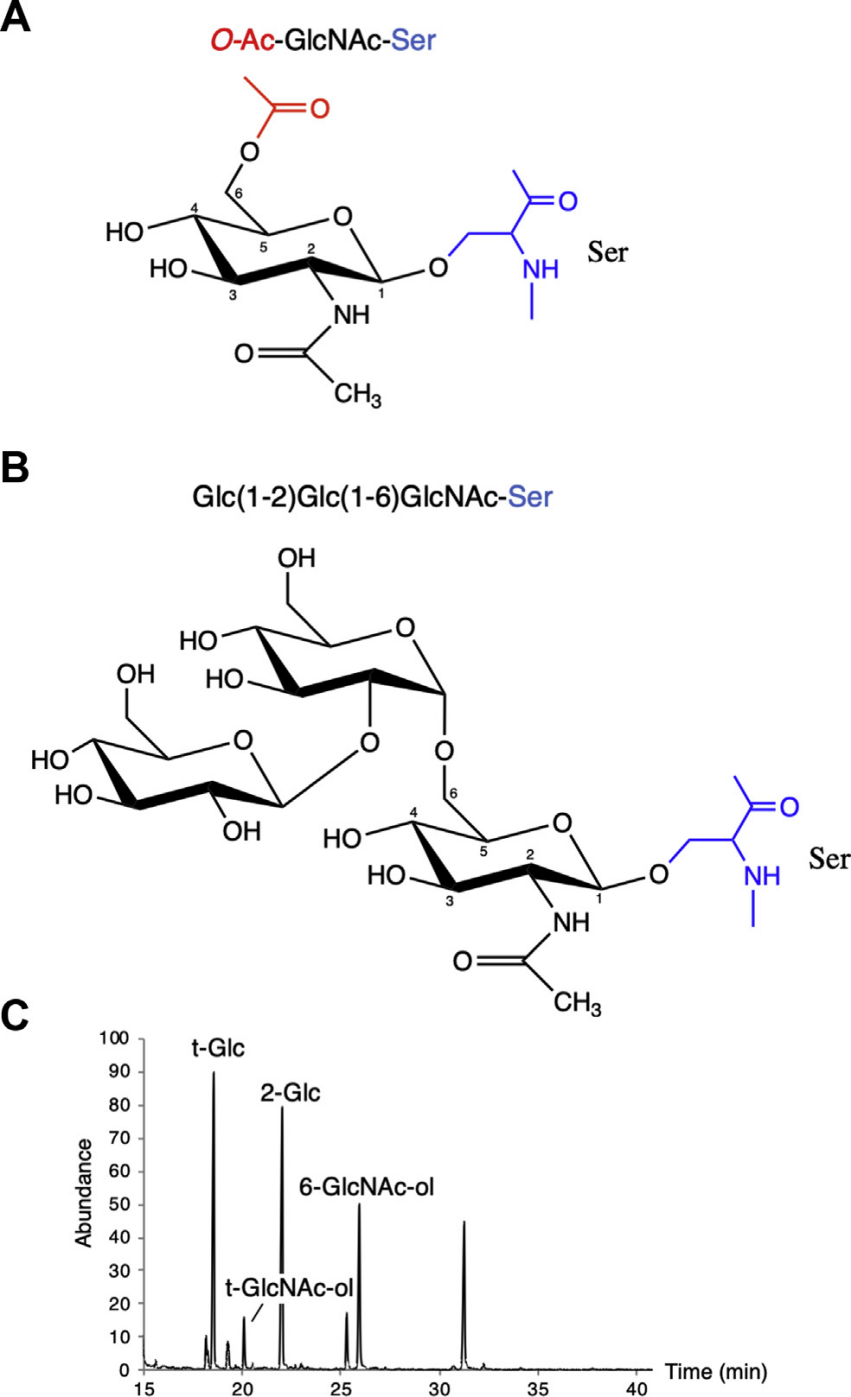
**Structures of *O*-acetylated and Nss and Gly modified glycans of GspB.***A,* The structure of *O*-acetylated GlcNAc was obtained from published glycopeptide CID fragmentation analysis of GspB and the SRR adhesin Srr1 of *S. agalactiae* (27). *B,* Structure of GlcNAc modified by both Nss and Gly was obtained by GC-MS profiling of partially methylated alditol acetates (PMAA) derived from glycans released from a recombinant GST-SRR protein co-expressed with GtfAB, Nss, and Gly. GlcNAc carbon positions are numbered. *C*, PMAA derivatives of the *O*-glycans released from GST-SRR1. Terminally linked glucose (t-Glc), terminally linked GlcNAc (t-GlcNAc), 2-linked glucose, and 6-linked GlcNAc were identified based on retention times in reference to carbohydrate standards. GspB, gordonii surface protein; GtfAB, glycosyltransferase complex; SRR, serine-rich repeat.

*O*-acetylation of the GspB glycan occurs at the C6-OH of core GlcNAc residues (27) (Fig. 3*A*). This is consistent with the location of the *O*-acetyl moiety on GlcNAc in the glycans of Srr1 of *S. agalactiae*, SrpA of *S. salivarius*, and *O*-acetylated forms of peptidoglycan (29, 30, 33, 34). In addition to *O*-acetylation, the GlcNAc core can be further modified through its sequential glycosylation by Gtfs such as Nss and Gly. To better assess how *O*-acetylation and glucosylation can simultaneously occur, we determined the structure of the glycans covalently attached to the SRR regions of GspB, by GC-MS analysis of partially methylated alditol acetates (PMAAs) or sugar linkage analysis of the GspB glycan.

To obtain sufficient quantities of the GspB glycan for linkage analysis, we used a recombinant GST–GspB fusion protein encompassing the SRR1 region, which undergoes glycosylation by all the Gtfs encoded within the *gspB-aSec* operon, generating a trisaccharide glycan (27). Analysis of the PMAA derivatives from the GspB glycan found the linkage of the trisaccharide to be Glc(1–2)Glc(1–6)GlcNAc (Fig. 3, *B* and *C*), where GlcNAc was identified as the reducing-end sugar. Resolution of the glycan structure revealed that Glc transferred by Nss is attached to GlcNAc at the C6 position, the same location that undergoes *O*-acetylation by Asp2. Collectively, these findings indicate that GlcNAc *O*-acetylation and glucosylation must be mutually exclusive events because they compete for the same site for GlcNAc modification.

### *O*-acetylation of GlcNAc directly blocks subsequent glucosylation

**Figure 4:**
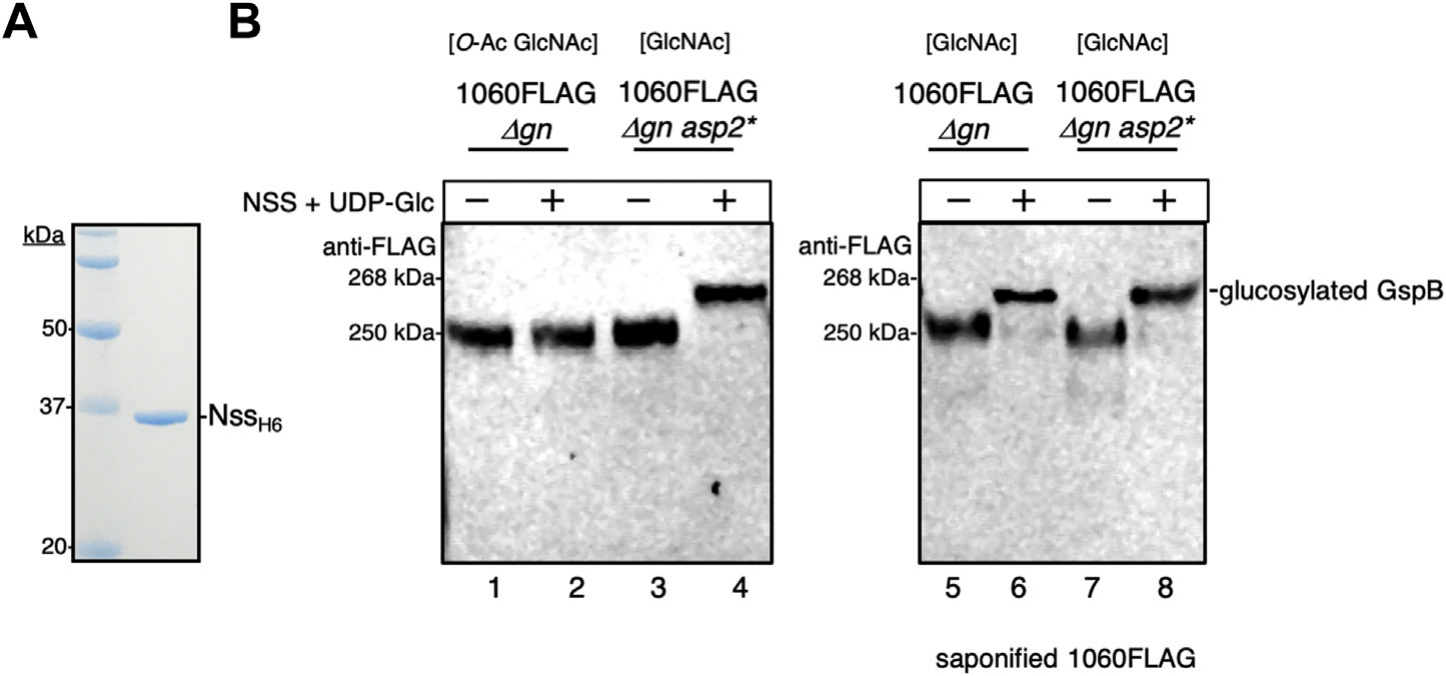
**Effect of *O*-acetylation on the subsequent glycosylation of GspB1060FLAG.***A*, SDS-PAGE analysis of purified NssH6 after expression in *E. coli* BL21 and purification on Ni-NTA resin. *B*, glucosylation of GspB1060FLAG before and after saponification. Acetylated (Δ*gn*) or nonacetylated (Δ*gn asp2*∗) GspB1060FLAG was combined with Nss_H6_ and UDP-Glc as indicated. Glucosylation reactions were carried out at 37 °C for 1 h and samples analyzed by SDS-PAGE and Western blot analysis with anti-FLAG antibodies. Samples were saponified by incubation with 100-mM NaOH for 1 h, to release *O*-acetyl moieties.

The finding that *O*-acetyl groups and Glc cannot modify the same GlcNAc residue, in conjunction with our observation that a loss of *O*-acetylation led to an increase in glucosylation of GspB, suggested that GlcNAc *O*-acetylation could serve to inhibit Glc deposition on GspB. To evaluate whether GlcNAc *O*-acetylation could control subsequent glucosylation of GspB, we tested whether *O*-acetylated and nonacetylated GspB glycoforms could be further modified by Nss *in vitro*. We purified the above glycoforms of GspB1060FLAG from M99Δ*gn* and M99Δ*gn asp2*^S362A^, respectively, mixed them individually with purified Nss and UDP-Glc, and assessed for the addition of Glc. *O*-acetylated GspB1060FLAG showed no obvious glucosylation, as indicated by no change in mass (Fig. 4*B*, lane 1 *versus* lane 2). In contrast, a 10-kDa increase in mass was seen with nonacetylated GspB1060FLAG after incubation with Nss and UDP-Glc, indicating that this glycoform was further modified by the addition of Glc to the GlcNAc core (Fig. 4*B*, lane 3 *versus* lane 4). Removing the *O*-acetyl groups on GspB1060FLAG by saponification before incubation with Nss and UDP-Glc produced a similar increase in mass (Fig. 4*B*, lane 5 *versus* 6, lane 7 *versus* 8), indicating further modification with Glc. These findings demonstrate that *O*-acetylation of GlcNAc residues prevents the subsequent Glc deposition on GspB. Moreover, the inability to detect *in vitro* glucosylation of exported WT GspB suggest GlcNAc residues are extensively *O*-acetylated in the WT glycan.

### Increased glucose deposition on GspB impairs platelet binding by bacteria

*O*-acetylation of the GspB glycan is essential for the binding of strain M99 to human platelets *via* this adhesin (27). Because *O*-acetylation inhibits glucosylation of GspB, we hypothesized that the loss of platelet binding associated with the *asp2*^S362A^ mutation could be due to increased glycosylation of GspB with Glc. To address this possibility, we directly compared platelet binding by M99 and isogenic variants that differed in their post-translational modifications of GspB.

**Figure 5:**
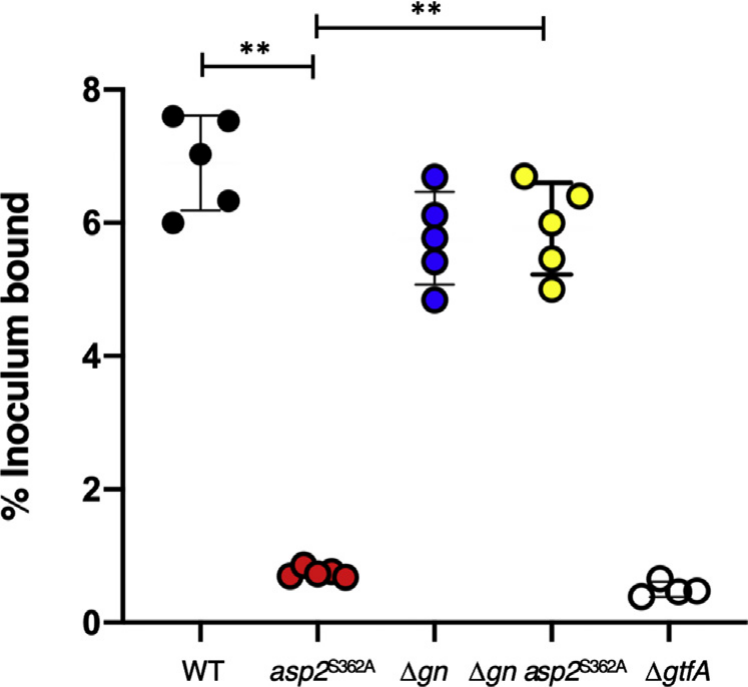
**Effect of *O*-acetylation on the binding of *Streptococcus gordonii* to human platelets.** WT strain M99 and mutants PS3536 (M99 *asp2*^S362A^), PS3319 (M99 Δ*gn*), PS3880 (M99 Δ*gn asp2*^S362A^), and PS666 (M99 Δ*gtfA*) expressing glycovariants of GspB were assessed for their binding to immobilized human platelets. Binding is expressed as the percent of input bacteria that remained bound to platelets after repeated washing of the wells (n = 5 + S.D. of triplicate results from a representative experiment). ∗∗*p* < 0.01, compared with WT M99 or PS3880 as indicated. Of note, no significant differences in platelet binding were seen between M99 and PS388.

As seen in previous studies (25), WT M99 exhibited high levels of binding to human platelets, while a M99Δ*gtfA* strain, which does not express GspB because of protein instability, exhibited low levels of binding (Fig. 5). Deletion of *gly* and *nss* produced only a small decrease in platelet binding (strain M99 *versus* M99Δ*gn*) that was not statistically significant. As expected, M99 expressing Asp2^S362A^ showed significantly reduced binding to platelets (*p* < 0.001 *versus* WT M99), to levels comparable to the Δ*gtfA* strain. However, deletion of *gly* and *nss* in combination with an *asp2*^S362A^ mutation (M99Δ*gn asp2*^S362A^) restored platelet binding to levels similar to those seen with the WT strain (*p* = 0.437). These results suggest that increased Glc deposition on GspB, and not the loss of the *O*-acetyl moiety, impairs the ability of GspB to bind to human platelets.

### Acetylation of SRR glycoproteins by Asp2 occurs in other species

**Figure 6:**
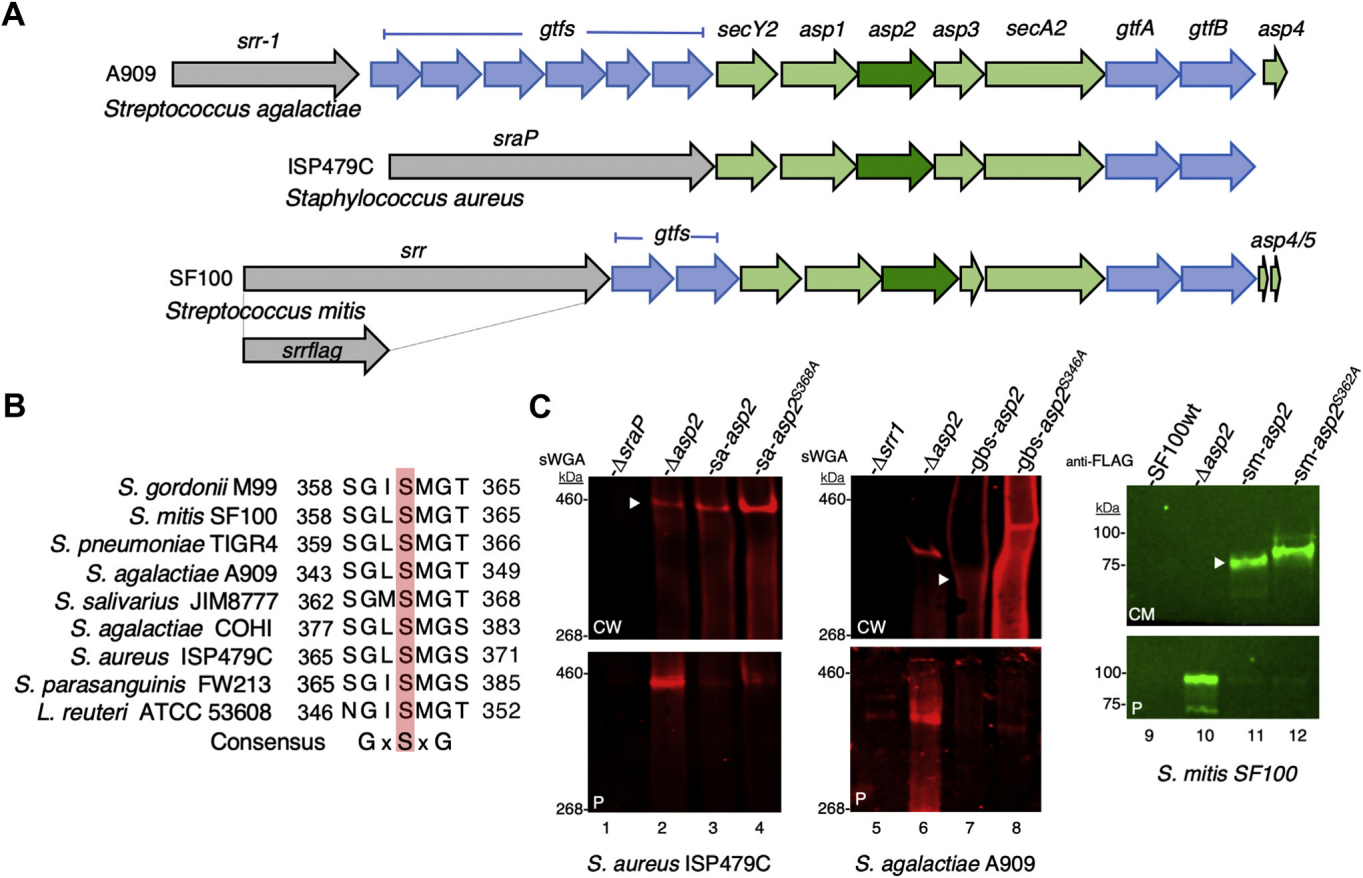
**Asp2 mediates the O-acetylation of SRR adhesins from other gram-positive pathogens.***A*, organization of the *srr-aSec* loci of strain M99 and the homologous regions of *S. agalactiae* strain A909, *Staphylococcus aureus* strain ISP479C, and *S. mitis* strain SF100. The genes encoding the SRR proteins are shown in *gray*, the aSec system components are in *green*, and the glycosyltransferases in *blue*. *B*, amino acid alignment of the conserved GxSxG motif in Asp2 homologues. The catalytic Ser residues are highlighted in *pink*. *C*, western blot analysis of full-length SRR proteins exported by *S. aureus* and *S. agalactiae* and truncated SF100-SRRFLAG expressed by *S. mitis* SF100. Cell wall (CW), culture medium (CM), and protoplast (P) fractions were prepared from strains in the exponential phase of growth. Proteins were separated by SDS-PAGE (3–8%) and analyzed by Western blotting using either biotinylated sWGA or anti-FLAG antibodies. *White arrows* indicate the position of the WT SRR glycoform. Asp2, aSec protein 2; SRR, serine-rich repeat.

Asp2 is an essential component of the aSec system (Fig. 6*A*) and is present in all bacteria expressing an SRR adhesin (8). Its acetyltransferase activity is mediated by a conserved Ser-Asp-His catalytic triad, where the serine residue is found within a GxSxG motif present in all Asp2 homologues (Fig. 6*B*). These findings suggest that other SRR glycoproteins undergo *O*-acetylation by Asp2. To test this possibility, we assessed the effect of mutating the catalytic site of Asp2 in *Staphylococcus aureus* ISP49C, *S. agalactiae* A909, and *Streptococcus mitis* SF100. Of note, each SRR adhesin is predicted to be glycosylated differently, based on the varying number of *gtfs* encoded within their respective *srr-aSec* operon (Fig. 6*A*). For each strain, we targeted the catalytic serine residue within the GxSxG motif of its Asp2 homologue (Fig. 6*B*) for alanine substitution. The resulting mutated *asp2* orfs were used to complement a variant of the parent strain containing an *asp2* deletion. Changes in the glycan of the SRR glycoproteins were assessed by sWGA binding and electrophoretic mobility.

Lectin blotting with sWGA of cell wall fractions from *S. aureus* ISP479C and *S. agalactiae* A909 revealed a single band in both WT strains that was lost with deletion of *sraP* or *srr1*, respectively (Fig. 6*C*, lane 1 *versus* 3 and lane 5 *versus* 7). Deletion of *asp2* in both WT strains reduced but did not abolish SRR glycoprotein export, with an intracellular accumulation of the preprotein in the Δ*asp2* deletion strains (Fig. 6*C*, lane 2 *versus* 3 and lane 6 *versus* 7). Complementation *in trans* of strains ISP49CΔ*asp2* and A909Δ*asp2* with their respective *asp2* variants sa-*asp2*^S368A^ and gbs-*asp2*^S346A^ resulted in the export of altered SraP and Srr1 glycoforms, (Fig. 6*C*, lane 3 *versus* 4 and lane 7 *versus* 8). Both SRR proteins showed increased binding to sWGA, and Srr1 migrated with an increase in mass, indicating a change to the SRR glycan. Of note, no differences in mass were seen between acetylated and nonacetylated SraP glycoforms (Fig. 6*C*, lane 3 *versus* lane 4), likely reflecting the lack of other *gtfs* within the *sraP-aSec* operon (Fig. 6*A*).

To more precisely assess the role of Asp2 in the glycosylation and export of SRR adhesins, we examined the extracellular transport of a truncated SRR glycoprotein (SRRFLAG) by *S. mitis* SF100. Deletion of *aps2* abolished SRRFLAG export, with a clear intracellular accumulation of the substrate (Fig. 6*C*, lane 10). Complementation with *asp2* led to the efficient export of SRRFLAG, where a ∼75 kDa protein was detected in the culture medium, and only trace amounts in the protoplasts. Similarly, complementation with sm-*asp2*^S362A^ restored glycoprotein export, but with SRRFLAG, now migrating with a higher molecular mass (∼85 kDa), indicating of loss of *O*-acetylation and increased glycosylation (Fig. 6*C*, lane 11 *versus* 12). Collectively, these findings demonstrate that the *O*-acetyltransferase activity of Asp2 is conserved in streptococci and staphylococci, and the loss of O-acetylation leads to the export of altered SRR glycoforms.

## Discussion

The SRR glycoproteins are a family of surface adhesins that are increasingly recognized as important for host colonization and virulence. Their biogenesis involves the intracellular glycosylation of the preprotein, followed by their transport to the bacterial surface by the aSec system (6, 8, 21). All SRR glycoproteins undergo the transfer of GlcNAc moieties to serine and threonine residues within the SRR domains (17). Analogous to the synthesis of mucins (35), the GlcNAc residues can be further modified by other Gtfs to generate a range of O-linked glycan structures, which can vary between species (6, 18, 20, 22). Changes in these O-linked glycans on SRR adhesins are correlated with impaired binding to host targets and biofilm formation, emphasizing the importance of glycosylation for optimal SRR glycoprotein function (4, 25).

Early reports on the glycans of Fap1 and PsrP examined the *in vitro* properties of the Gtfs that modified theses adhesins, using recombinant SRR proteins as Gtf substrates, or by coexpressing the Gtfs and adhesins in *Escherichia coli* (20, 21). These studies were key in determining the specific enzymatic functions of the Gtfs and in identifying the glycan structures of these adhesins (20, 21). More recent direct analysis of the native glycan of the SRR glycoproteins Srr1 and SrpA revealed heterogeneity of the glycan, where not all of the GlcNAc core is further modified (29, 30), indicating that the glycan is not fully extended at all glycosylation sites under normal conditions.

An additional modification of the SRR adhesins is the *O*-acetylation of GlcNAc residues at the C6 hydroxyl position by Asp2 (27, 29, 30). Our results indicate that for GspB, this is the same position that can be further glucosylated to generate the Glc(1–2)Glc(1–6)GlcNAc trisaccharide. *O*-acetylation and glucosylation compete for the same site to modify GlcNAc residues, such that *O*-acetylation can block glucosylation of the GlcNAc core. Thus, GlcNAc *O*-acetylation is a regulatory modification preventing high levels of glucosylation of the GspB SRR domains, which can hinder ligand binding. Although we did not examine this directly, the blocking of glucosylation of the GlcNAc core by *O*-acetylation could also serve as a mechanism for generating glycan heterogeneity throughout the SRR regions of GspB.

Limiting the glycosylation of the GlcNAc core had a profound effect upon the binding properties of GspB. We have previously shown that the loss of *O*-acetylation markedly reduced GspB-mediated streptococcal binding to human platelets, to levels comparable with a *gspB*-deletion strain (27). Our finding that the deletion of *gly* and *nss* in an *O*-acetylation mutant restored platelet-binding indicates that the reduced binding is not directly due to the loss of acetylation but instead stems from the additional glucosylation of GlcNAc. How increased glucosylation of the GspB SRR domains can adversely affect platelet-binding function remains unclear. High levels of glucosylation of the SRR regions could either induce a conformation change within the BR or occlude its access to the GPIbα receptor on platelets. We recently found that, when tested under flow conditions, streptococcal binding to immobilized platelet GPIbα increased with shear force (36). This finding suggests that under static conditions, the flanking glycosylated SRR regions may occlude the BR, whereas under shear force, the SRR regions undergo a conformational change that allows the BR to interact with GPIbα (36). If this model proves to be correct, increased glucosylation may hinder the flexibility of GspB, preventing subsequent presentation of the BR domain to the host receptors.

**Figure 7:**
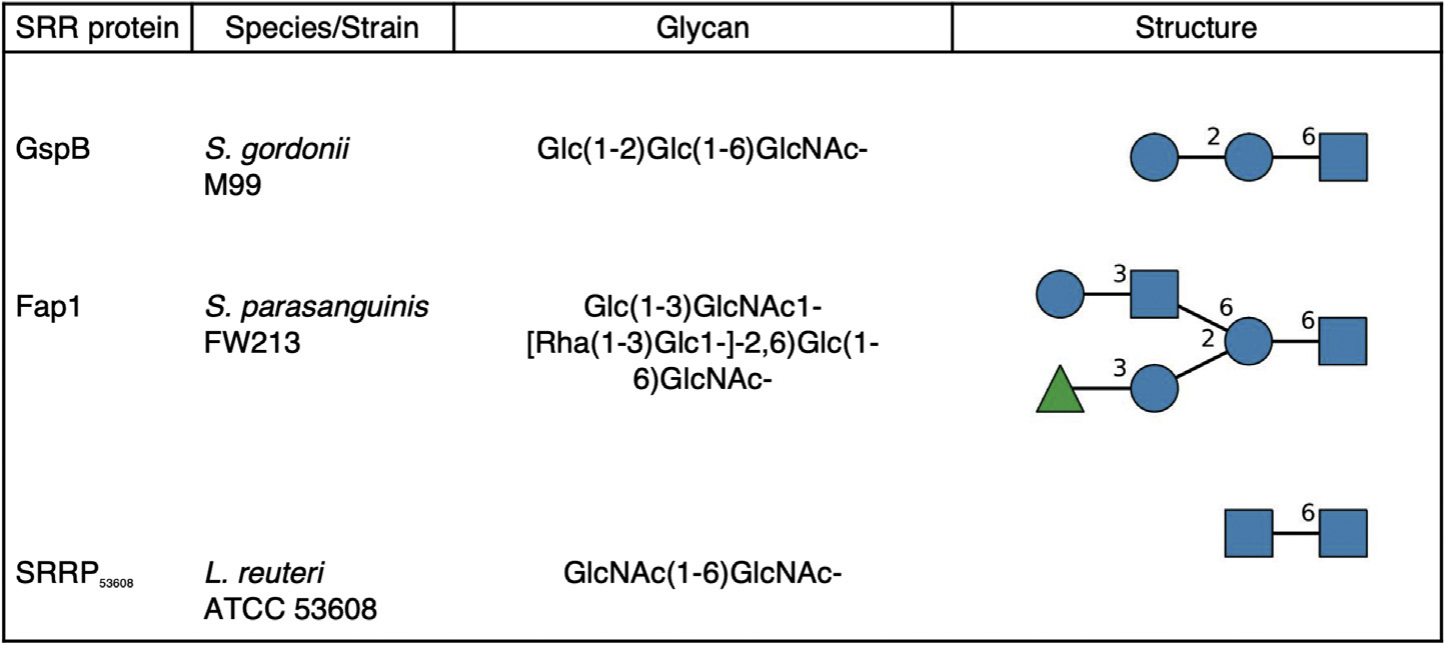
**Determined glycan linkages from known SRR protein glycan structures.** Glycan structures of Fap1 and SRRP_53608_ as reviewed by Latousakis *et al.* (6). Monosaccharide symbols follow the Symbol Nomenclature for Glycans system (50). GlcNAc (*blue square*), glucose (Glc: *blue circle*), rhamnose (Rha; *green triangle*).

The ability to regulate further glycosylation of the GlcNAc core *via O*-acetylation may be a common mechanism in the biogenesis of SRR adhesins. As detailed above, catalytic mutants of Asp2 in *S. agalactiae*, *S. mitis*, and *S. aureus* all had altered glycosylation of their respective SRR adhesins, indicating that Asp2-mediated *O*-acetylation is a highly conserved regulatory mechanism for the post-translational modification of these adhesins. Moreover, analysis of the glycan of Fap1 of *S. parasanguinis* and SRRP_53608_ of *Lactobacillus reuteri* revealed that their glycans were also attached to core GlcNAc *via* the C-6 position (Fig. 7), indicating that *O*-acetylation of these SRR adhesins also serves to regulate modification of the GlcNAc core. Of note, the glycan of SraP from *S. aureus* is composed of GlcNAc monosaccharides (37), suggesting that *O*-acetylation may have additional roles in SRR glycoprotein function.

*O*-acetylation of bacterial glycans have been shown to control the deposition of additional sugars in other bacterial species. O-antigen of the lipopolysaccharide of *Shigella flexneri* consists of a tetrasaccharide [-GlcNAc(1–2)Rha(1–2)Rha(1–3)Rha-] repeat that can be further modified by bacteriophage-encoded glucosyltransferase and *O*-acetyltransferases, generating antigenic determinants that define serotype specificity (38, 39). Interestingly, the introduction of *oacD* (the acetyltransferase-mediating O-antigen GlcNAc O-acetylation) *via* integration of bacteriophage SfII abolished prior GlcNAc glucosylation at the C-6 position, converting the O-antigen serotype (40). These findings, in combination with our own on GspB glucosylation, suggest that GlcNAc *O*-acetylation may serve to prevent further GlcNAc modification at the C6 position for a broad range of bacterial glycans.

**Figure 8:**
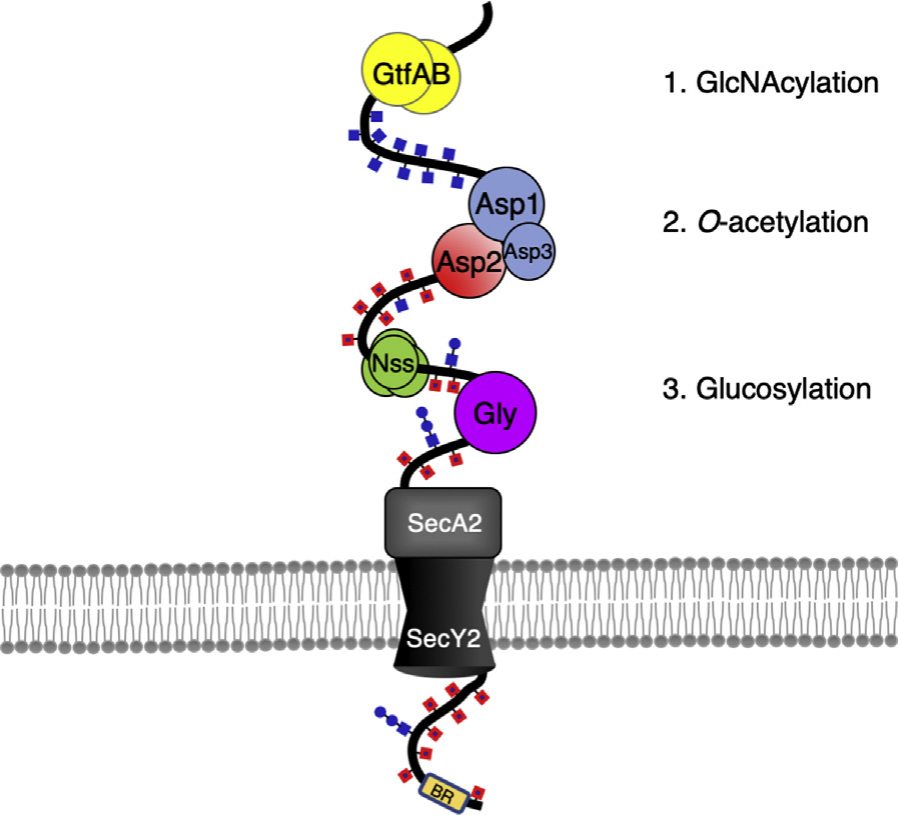
**Model for GspB glycoprotein biogenesis.** GspB is sequentially glycosylated by the GtfAB complex that deposits GlcNAc along the SRR region. GlcNAc-modified GspB engages with the aSec system where upon GlcNAc residues are *O*-acetylated. Before full engagement with SecA2 at the cell membrane, GspB is further modified by Nss and Gly. GtfAB, glycosyltransferase AB complex; GspB, gordonii surface protein B; SRR, serine-rich repeat.

The finding that *O*-acetylation of GlcNAc by Asp2 controls glucose deposition suggests that Asp2 must engage with the SRR preprotein before Nss and Gly. How *O*-acetylation is prioritized over glucosylation is unknown. We have shown previously that *O*-acetylation by Asp2 is coupled to aSec transport (27), although it is unclear how these two processes could be coordinated. One possibility is that signal peptide–mediated targeting of SRR adhesins to the aSec translocon brings the preprotein into contact with all aSec components, thereby facilitating *O*-acetylation by Asp2. Such substrate targeting could serve to prioritize *O*-acetylation after GtfAB glycosylation during aSec export. In addition, Asp2 forms a complex with Asp1 and Asp3 that is necessary for GspB export (18, 41). Although a direct role of Asp1 and Asp3 in O-acetylation has not been demonstrated, it is conceivable that the Asps are also involved in coordinating aSec transport and *O*-acetylation of SRR glycoproteins (Fig. 8).

Our findings provide new insights into the importance of *O*-acetylation and Asp function in the biogenesis of SRR glycoproteins. Although it was previously proposed that Nss and Gly glucosylation occurred shortly after GtfAB glycosylation (42), our data indicate that GlcNAc *O*-acetylation modulates subsequent Glc addition. Therefore, we propose a revised model of SRR biogenesis, where the SRR preprotein is modified with GlcNAc by GtfAB, followed by Asp-mediated *O*-acetylation of the SRR glycan before further glycosylation by additional Gtfs and export through the aSec translocon (Fig. 8). Delineating the consequences of these glycan changes in relation to aSec component engagement will be key to better understanding the need for a dedicated SRR export system.

## Experimental procedures

### Bacterial strains and plasmids

The bacterial strains and plasmids used in this study are listed in Table 1. *S. gordonii*, *S. agalactiae*, and *S. mitis* strains were grown in the Todd-Hewitt broth (Becton, Dickinson and Company) or on 5% sheep blood agar (Hardy Diagnostics) at 37 °C in a 5% CO_2_ environment. *S. aureus* strains were grown at 37 °C under aeration in the Tryptic soy broth (Becton, Dickinson and Company). For some experiments, antibiotics were added to the media at the following concentrations: 50 μg/ml erythromycin and 100 μg/ml spectinomycin for *S. gordonii* and *S. mitis* strains; 15 μg/ml erthromycin for *S. aureus* strains; 5 μg/ml erythromycin for *S. agalactiae* strains; 5 μg/ml chloramphenicol for *S. mitis* and *S. agalactiae* strains. *E. coli* strain TOP10 was grown in LB broth or on LB agar containing 50 μg/ml kanamycin, 300 μg/ml erythromycin, 50 μg/ml spectinomycin and 15 μg/ml chloramphenicol when appropriate.

### Construction of M99 strains expressing Asp2 catalytic mutants

An *asp2*^S362A^ point mutation was introduced into *S. gordonii* strain M99 through allelic replacement as previously described (27). In brief, plasmid pCOLA_H6_*asp123* (encoding *asp123*) containing a Ser-362-Ala codon replacement within *asp2* was used as a template for heterologous recombination within the genome of M99 Δ*asp2::spec* strains. Plasmids were used to transform M99 Δ*asp2::spec* strains by natural transformation resulting in a replacement of a spectinomycin cassette with the mutated *asp2* orf. Transformants were plated on sheep blood agar plates and scored for the loss of spectinomycin resistance. Chromosomal DNA was isolated from spectinomycin-sensitive clones according to Madoff *et al.* (43), and the *asp2* gene was PCR-amplified and sequenced to confirm the correct mutation had occurred.

### Deletion of *gly* and *nss*

Deletion of *gly* or *nss* genes in the *gspB*-*aSec* locus of M99 variants was performed by allelic replacement as previously described (25).

### Construction of *S. mitis* strains expressing variants of SRRFLAG

A variant of *S. mitis* SF100 expressing a FLAG-tagged version of its SRR adhesin was constructed as described previously for *S. gordonii* M99 expressing GspB736FLAG (26). In brief, the suicide vector p736BflagC containing codons 604 to 736 of the GspB SRR2 region fused to a 3xFLAG sequence was used to transform *S. mitis* SF100 by natural transformation as described previously (44). The resulting strain (PS3397), encoding the SF100 SRR protein with a truncated SRR2 region and C-terminal 3xFLAG tag, was then used to express variant forms of SRRFLAG in *S. mitis* strain SF100.

### Deletion of asp2 in *S. mitis* and *S. agalactiae*

To generate an *asp2* deletion in either *S. mitis* SF100 or *S. agalactiae* A909, a gene replacement cassette was constructed by cloning the chromosomal regions flanking *asp2* upstream and downstream of the chloramphenicol (*cat*) gene in pC326 (45). The resulting plasmids, pSF100*asp2*-KO and pA909*asp2*-KO, were introduced into the above strains by natural transformation and electroporation, respectively (44, 46). *asp2*-deletion mutants were selected by plating onto blood agar containing chloramphenicol.

### Asp2 complementation

*asp2* homologues were amplified by PCR from the genome of *S. aureus* ISP479C, *S. agalactiae* A909, or *S. mitis* SF100 and cloned as N-terminal 6xHis-tag fusions into the expression plasmids pKS80 (47) or pDE123 (44). Plasmids were introduced into staphylococcal and streptococcal strains by electroporation, as described previously (46, 48).

### Analysis of secreted cell wall and protoplast proteins

Overnight cultures of *S. gordonii*, *S. mitis*, and *S. agalactiae* were diluted 1:6 in fresh Todd-Hewitt broth, grown at 37 °C, and cells were harvested at mid-logarithmic growth by centrifugation. For analysis of secreted proteins, samples of clarified culture media were mixed with protein sample buffer (Novagen) before SDS-PAGE separation and Western blot analysis or used directly for saponification analysis. For analysis of cell wall proteins, pelleted cells were resuspended in spheroplast buffer (60-mM Tris, pH 7, 150-mM NaCl, 25% raffinose, and 0.5 U/μl mutanolysin). The suspensions were incubated for 1 h at 37 °C and centrifuged at 10,000*g* for 10 min at room temperature (RT). The supernatants containing the cell wall extracts were mixed and boiled in protein sample buffer, followed by SDS-PAGE and Western blot analysis. The remaining pellets containing protoplasts were resuspended and lysed in protein sample buffer before gel electrophoresis. Preparation of cell wall and protoplast extracts from *S. aureus* were carried out as described previously (48). Anti-FLAG and anti-GspB antibodies were detected using fluorescent anti-mouse IRDye 800 and anti-goat IRDye 800 secondary antibodies, respectively. sWGA was detected with streptavidin IRDye 680 (red). Immunoreactive bands were visualized using the LI-COR Odyssey Infrared Imaging System.

### Linkage analysis of glycosylated GST-SRR1

Glycan linkages were determined through the GlycoAnalytics Core center at the University of California, San Diego. Glycans were liberated from purified GST-SRR1 glycosylated by GtfAB, Nss, and Gly by reductive beta elimination using base-borohydride treatment (27). The *O*-glycans were then purified and partially methylated according to the method of Ciucannu and Kerek (49). *O*-glycans were dried and dissolved in anhydrous dimethyl sulfoxide, followed by adding NaOH slurry in dimethyl sulfoxide and methyl iodide. Partially methylated glycans were extracted by partitioning in water–chloroform mixture, and the chloroform layer was dried and used for linkage analysis. The partially methylated glycans were hydrolyzed using 4 N TFA at 100 °C for 6 h, followed by removal of the acid, reduced with sodium borodeuteride and acetylated to generate the PMAA derivatives, which were then analyzed by GC-MS using Restek-5ms capillary column for inter-residue linkages. Identifications are achieved by using a combination of retention times and electron ionization mass fragmentation pattern.

### Cloning, overexpression, and purification of Nss

*nss* was cloned as a C-terminal 6xHis-tag fusion within the expression plasmid pET28b (Novagen). The pET*nss* construct was introduced to *E. coli* BL21 (Lucigene), and expression was induced by the addition of 1-mM IPTG. Nss_H6_ was purified from clarified lysates under native conditions by Ni-NTA–affinity chromatography (Qiagen). Purified Nss_H6_ was concentrated by ultrafiltration using an Amicon Ultra centrifugal filter (10 kDa cutoff) and dialyzed against 25-mM Tris, pH 7, and 150-mM NaCl overnight at 4 °C before use.

### *In vitro* glucosylation assays

GspB1060FLAG secreted from M99Δ*gn* or M99Δ*gn asp2*^S362A^ served as a substrate for Nss *in vitro* glucosylation of GspB. Cultures of each strain were grown to the mid-log phase, and 15 ml of the culture media were concentrated 50-fold by ultrafiltration as described above and reconstituted in 50-mM Tris, pH 7. Some samples of GspB1060FLAG in culture media were saponified as described below, before concentration and reconstitution. *In vitro* glucosylation assays were performed at 37 °C for 1 h. To initiate glucosylation of GspB1060FLAG, 10 μM of Nss and 20-mM UDP-Glc (Sigma) were mixed with 15 μl of GspB1060FLAG solution in 50-mM Tris, pH 7, with a final reaction volume of 20 μl.

### GspB saponification

Removal of glycan *O*-acetyl groups was achieved by base-promoted ester hydrolysis. Culture supernatants containing secreted GspB variants were buffered in 10-mM Tris, pH 9, and incubated with 100-mM NaOH for 1 h at 37 °C.

### Binding of *S. gordonii* to platelet monolayers

The binding of *S. gordonii* to immobilized platelets was performed as described previously (15). In brief, strains were grown for 18 h, washed twice in Dulbecco's PBS (DPBS), sonicated briefly to disrupt aggregated cells and diluted to approximately 2 × 10^7^ cfu/ml. Bacterial suspensions were applied to wells of a microtiter plate coated with human platelets and blocked with a casein solution [blocking reagent (Roche) in DPBS] to prevent nonspecific bacterial adherence. After 2-h incubation at RT, the unbound bacteria were removed by aspiration. Wells were washed three times with DPBS, and the bound bacteria were released by trypsin treatment (1-h incubation with 50 μl of 1 mg/ml trypsin at RT). The number of input and bound bacteria was determined by plating serial dilutions of bacterial suspensions on sheep blood agar plates, and the binding was expressed as the percent of the input bound to human platelets. Differences in binding were compared by one-way ANOVA with post hoc Tukey's honestly significant difference (https://astatsa.com/OneWay_Anova_with_TukeyHSD/). *p* < 0.01 was considered statistically significant.

## Data availability

Data sets associated with GC-MS linkage analysis of the GspB trisaccharide, together with MS spectra of the partially methylated alditol acetate (PMAA) derivatives, reported here have been deposited in the publicly accessible repository figshare (https://doi.org/10.6084/m9.figshare.13198340.v2). All other data are available in the manuscript.

## Conflict of interest

The authors declare that they have no conflicts of interest with the contents of this article.
